# Dealing with prognostic signature instability: a strategy illustrated for cardiovascular events in patients with end-stage renal disease

**DOI:** 10.1186/s12920-016-0210-9

**Published:** 2016-07-20

**Authors:** Harald Binder, Thorsten Kurz, Sven Teschner, Clemens Kreutz, Marcel Geyer, Johannes Donauer, Annette Kraemer-Guth, Jens Timmer, Martin Schumacher, Gerd Walz

**Affiliations:** Institute of Medical Biostatistics, Epidemiology and Informatics, University Medical Center Mainz, Obere Zahlbacher Str. 69, Mainz, 55131 Germany; Core Facility Genomics, Centre for Systems Biology, University Freiburg, Freiburg, Germany; Renal Division, Department of Medicine, University Hospital Freiburg, Freiburg, Germany; Institute of Physics, University Freiburg, Freiburg, Germany; Dialysis Freiburg & Breisach, Freiburg, Germany; Dialysis Center Freiburg, Freiburg, Germany; Institute of Medical Biometry and Medical Informatics, University Medical Center Freiburg, Freiburg, Germany; BIOSS Center for Biological Signalling Studies, University Freiburg, Germany, Freiburg, Germany

**Keywords:** Prognostic signature, Stability, Clinical endpoint, Outlier

## Abstract

**Background:**

Identification of prognostic gene expression markers from clinical cohorts might help to better understand disease etiology. A set of potentially important markers can be automatically selected when linking gene expression covariates to a clinical endpoint by multivariable regression models and regularized parameter estimation. However, this is hampered by instability due to selection from many measurements. Stability can be assessed by resampling techniques, which might guide modeling decisions, such as choice of the model class or the specific endpoint definition.

**Methods:**

We specifically propose a strategy for judging the impact of different endpoint definitions, endpoint updates, different approaches for marker selection, and exclusion of outliers. This strategy is illustrated for a study with end-stage renal disease patients, who experience a yearly mortality of more than 20 %, with almost 50 % sudden cardiac death or myocardial infarction. The underlying etiology is poorly understood, and we specifically point out how our strategy can help to identify novel prognostic markers and targets for therapeutic interventions.

**Results:**

For markers such as the potentially prognostic platelet glycoprotein IIb, the endpoint definition, in combination with the signature building approach is seen to have the largest impact. Removal of outliers, as identified by the proposed strategy, is also seen to considerably improve stability.

**Conclusions:**

As the proposed strategy allowed us to precisely quantify the impact of modeling choices on the stability of marker identification, we suggest routine use also in other applications to prevent analysis-specific results, which are unstable, i.e. not reproducible.

## Background

Several studies have established the association between chronic kidney disease (CKD) and cardiovascular disease (CVD), including sudden death, stroke, congestive heart failure and myocardial infarction [1]. Once patients reach end-stage renal disease (ESRD) and require renal replacement therapy, the yearly mortality exceeds 15–20 % [2]. More than 50 % of patient deaths are caused by cardiovascular events [3, 4], and the two-year mortality rate after myocardial infarction among patients with ESRD is twice the mortality rate compared to myocardial infarction in the general population [5]. Cardiovascular disease in ESRD has largely been attributed to the significant co-morbidity such as advanced age, hypertension and diabetes mellitus, i.e. conditions often associated with both chronic renal failure and cardiovascular disease. However, these clinical risk factors alone cannot explain the high incidence of cardiovascular events particularly in younger ESRD patients. To obtain further insight in the etiology of cardiovascular disease in ESRD, we investigated the link between the gene expression profiles of 321 hemodialysis patients and the incidence of cardiovascular events over a two-year observation period.

There are many issues that can affect performance of prognostic models in such an application, such as the measurement platform (e.g. microarrays vs. RNA-seq [6]), the type of sample (e.g. fresh frozen vs. paraffin embedded [7]), and early preprocessing steps [8]. In the following, we focus on issues arising in subsequent modeling steps. The ESRD application has several features that make it interesting for illustrating a stability strategy that we propose for prognostic signature development. First, the number of events of interest, i.e. the cardiovascular events, will be relatively small even for the relatively large cohort considered here. This makes it challenging to develop a stable prognostic signature. Specifically, the set of genes to be selected for a signature will vary considerably when signatures would be developed repeatedly in different cohorts, even if reasonable prediction performance is obtained in each single cohort [9]. As larger cohorts or longer follow-up (for increasing the number of observed events) might not be feasible, we propose a strategy for identifying factors affecting signature stability in the cohort at hand. A second challenge arises from the complex time-to-event endpoint structure. As discussed in more detail in Section “Material and methods”, it is difficult to determine the exact time point for cardiovascular events not leading to death during an observation period, only the endpoint “death after cardiovascular event” can be considered in our application. In addition, there is the competing event/risk “death without prior cardiovascular event”. This leaves at least two options for modeling the time-to-event endpoint of interest, either via the cumulative incidence of death after cardiovascular events, i.e. the observed proportion in the course of time, or the cause-specific hazard [10, 11]. As a third challenge, there are actually two endpoint definitions in the present application, the original endpoint information, and revised endpoint information from an update performed at a later time. As such endpoint updates are typical for cohorts with longer follow-up, it will be interesting to judge the potential effect of these on signature stability, in particular relative to the effects of different bioinformatic approaches for signature building. For the latter, we consider univariate gene selection vs. a multivariable regularized regression approach [10].

**Fig. 1:**
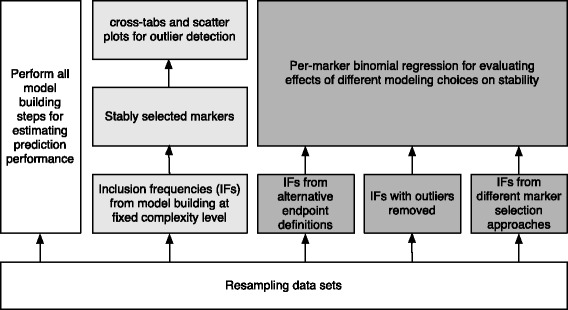
Strategy building blocks for investigating and improving signature stability. All steps are based on resampling data sets, which serve as a foundation. Subsequent steps involving all model building steps, e.g. cross-validation for model complexity selection, are indicated in white, steps based on fixed complexity levels in gray. Of the latter, steps for identifying outliers are indicated by *light gray*, steps for investigating different modeling strategies are indicated by *darker gray* shading

The building blocks of the proposed strategy for judging effects on stability are illustrated in Fig. 1. The basis for all steps is provided by resampling techniques. Stability will be quantified by resampling inclusion frequency, which has already been suggested some time ago [12–14], but has recently received renewed attention due to stability path approaches that potentially allow to control false discovery rates [15, 16]. We will specifically build on the idea of cross-tabulation of inclusion frequencies [14] for identifying outliers, and subsequently quantify the effect of such outliers relative to the factors influencing stability indicated above. This is particularly important, as outliers might not only severely hamper analysis of microarray data (as in the present application), but can also drastically impact performance of signatures based on RNA-seq measurements [17].

The effects of different modeling decisions on resampling inclusion frequencies, i.e. selection stability, will be quantified by regression models and contrasted to prediction performance of different models. This will highlight what can be gained by moving focus from prediction performance to stability for judging the reliability of potential etiological insight. We will also consider selection (in-)stability for one specific marker (platelet glycoprotein IIb), which we identified and deemed interesting in a first analysis, for indicating potentially detrimental effects of specific modeling choices on selection of interesting markers that might have only moderate effect.

## Methods

### Study design and population

This study was designed to identify a potential link between the gene profiles of circulating blood cells of hemodialysis patients and the occurrence of cardiovascular events over a 2-year observation period. The institutional ethics committee at the University Hospital Freiburg approved the protocol; the study was conducted in accordance with the Declaration of Helsinki at four outpatient hemodialysis centers in Germany. After obtaining informed consent, blood samples were collected from 324 hemodialysis patients immediately before dialysis treatment following a two-day dialysis-free interval; 3 samples were excluded due to poor RNA quality, the remaining 321 samples were processed as outlined below. Nineteen covariates, including age, sex, duration of dialysis, and previous cardiovascular events, were recorded at the time of enrollment; clinical chemistry, including lipid profile and hematological parameters, were extracted from the patients’ records (Table 1). Patients were subsequently followed for two years. As we could not directly observe the time of cardiovascular events, patients were monitored for two other types of events that allow for an indirect link of gene profiles to cardiovascular events: We monitored for death, an used patient records for identifying whether a patient had a cardiovascular event prior to death (without requiring a casual link). Thus, we effectively monitored patients for “death with prior cardiovascular event” and “death without prior cardiovascular event”.

**Table 1:** Clinical data of 321 ESRD patients on chronic intermittent hemodialysis

Patients	
Age (yrs)	66.3 (± 17.3)
Sex (Male (%) / Female (%))	197 (61) / 124 (39)
BMI (kg/m ^2^)	25.2 (± 5.1)
Dialysis	
Time on dialysis at inclusion (months)	49 (± 46)
Dialysis treatment duration per week (hrs)	12.7 (± 2.5)
Kt/V (mean of last three	1.27 (± 0.34)
measurements before inclusion)	
Renal disease	
Diabetic nephropathy (%)	85 (26)
Glomerulonephritis (%)	75 (23)
Hypertensive/Vascular (%)	48 (15)
ADPKD (%)	26 (8)
Other (%)	46 (14)
Unknown (%)	41 (13)
Clinical chemistry	
Total cholesterol (mg/dL)	177 (± 43)
Triglycerides (mg/dL)	197 (± 145)
Hemoglobin (g/dL)	11.7 (± 1.5)
CRP (mg/L)	30.5 (± 71.5)
Urea before dialysis (mg/dL)	121 (± 43)
Phosphate (mean of three predialysis	5.6 (± 1.7)
measurements, mg/dL)	
Total Calcium (corrected for albumin (mmol/L))	2.3 (± 0.3)
Calcium × Phosphate product	50 (± 14)
Parathormon, in patients not	158 (± 201)
parathyroidectomized (pg/ml)	
Ferritin (ng/mL)	540 (± 332)
Albumin (g/dl)	4.0 (± 0.6)

BMI: Body mass index; ACE-I: Angiotensin-converting enzyme inhibitor; ARB: Angiotensin receptor blocker; MI: Myocardial infarction

### Time-to-event regression models

In the following we briefly sketch the theory of the hazard-based time-to-event regression models in a competing risks setting, before discussing their use in the present application (for details see, e.g., [11]). Observations are given as (*T*_*i*_,*Δ*_*i*_*ε*_*i*_,*X*_*i*_),*i*=1,…,*n*, where *T*_*i*_ is the observed time, *Δ*_*i*_ is a censoring indicator taking value 1 if an event has been observed at time *T*_*i*_ and value 0 otherwise, and *ε*_*i*_∈{1,…,*K*} is the event type. The covariate vector *X*_*i*_=(*X*_*i*1_,…,*X*_*ip*_)^′^ contains clinical covariates as well as potential molecular markers, such as gene expression measurements.

For investigating effects of the latter on an event type of interest, e.g. *ε*=1, the cause-specific hazard

$$h_{cs1}(t) = {\lim}_{\delta \to 0} \frac{P(t\leq T < t + \delta, \epsilon = 1)}{\delta} $$

can be linked to the covariates via a Cox proportional hazards model

$$h_{cs1}(t|X) = h_{0,cs1}(t)\exp(X'\beta), $$

where *h*_0,*cs*1_(*t*) is an unspecified baseline hazard, and *β* is a parameter vector of length *p*, each element specifying the influence of one clinical covariate or molecular marker.

Alternatively, the cumulative incidence

$$F_{1}(t) = P(T < t, \epsilon = 1) $$

can be considered for analysis. Specifically, the Fine-Gray model

$$h_{sh1}(t|X) = h_{0,sh1}(t)\exp(X'\beta) $$

has been suggested for analyzing effects on the subdistribution hazard

$$h_{sh1} = \frac{dF_{1}(t)/dt}{1-F_{1}(t)}, $$

which is directly linked to the cumulative incidence [18].

In the present application, the time of cardiovascular events could not be observed exactly. Therefore, an indirect analysis was performed with Cox models for the events “death with prior cardiovascular event” and “death without prior cardiovascular event”. We differentiated between the states (1) “patient alive without cardiovascular event”, (2) “patient alive with cardiovascular event”, (3) “death after cardiovascular event”, and (4) “death without prior cardiovascular event”, where in state (3) the cardiovascular event does not necessarily have to be the cause of death. While the exact time points for reaching state (3) or (4) can be recorded, this is not the case for the transition between (1) and (2). However, each patient entering state (3) has to pass through state (2). Therefore, the cumulative incidence of (3), i.e. the proportion of observed deaths after a cardiovascular event in the course of time, is the cumulative incidence for (2) multiplied by the probability of dying of any cause. If a gene is linked to (3), this could either be due to a linkage to (2), or due to a linkage to death in general. To distinguish between these two possibilities, we considered two types of statistical models. The first model investigated the connection between gene expression and the cumulative incidence for state (3). The second model linked gene expression to the risk for reaching state (4). If a gene had an effect only in the first type of model, but not in the second, it had to be linked to the transition from state (1) to state (2), but not to the transition between (2) and (3). Therefore, the gene had to be linked to cardiovascular events. As an alternative we consider cause-specific hazard models for the event “death after cardiovascular event”.

### Componentwise boosting

To identify differentially regulated genes linked to future cardiovascular events, all genes were considered simultaneously in a multivariable model that takes correlations between genes into account. Specifically, we used componentwise likelihood-based boosting [10] for estimating the parameter vectors in the regression models above. By performing regularized estimation, i.e., forcing estimates towards zero, this boosting approach not only performs variable selection, but also allows to select a relatively large number of genes for a risk prediction signature with an effective number of parameters that is much smaller than the number of included genes, thus avoiding overfitting. Briefly, this is achieved by starting with an estimated parameter vector equal to zero, and updating its elements in a potentially large number of boosting steps. In each step only one element is updated (by a shrunken maximum partial likelihood estimate), chosen to to maximize the partial likelihood. The number of boosting steps in the present application was either chosen by 10-fold cross-validation, when considering prediction performance, or a fixed number of 100 boosting steps was used for stability analyses.

In the present analysis, age and prior cardiovascular events were included as mandatory covariates to compare the prediction performance of this approach with a purely clinical model that incorporated only the clinical covariates. Componentwise likelihood-based boosting is one of the few approaches that has been adapted for competing risks setting with a readily available implementation, and at the same time allows for inclusion of such mandatory covariates. Furthermore, it is computationally fast compared to other high-dimensional multivariable approaches, which is advantageous when large numbers of repeated model fits have to be performed, as in the present application.

To adjust for effects of other differences in patient characteristics, the following baseline clinical covariates were initially considered as candidates for model inclusion, i.e. given the chance for selection just as the gene expression measurements: gender, smoking, adiposity, low HDL cholesterol, hypercholesterolemia high CRP, high homocysteine level, prior coronary artery disease, arterial hypertension, diabetes, diabetic nephropathy, secondary hyperparathyroidism, parathyroidectomy, acidosis, hyperuricemia, duration of dialysis and family history of cardiovascular disease. However, as these were not selected by componentwise boosting, i.e. they do not seem to have a strong effect, we did not consider them for subsequent analyses.

Model fitting as well as estimation of prediction performance was performed in the statistical environment R (version 3.1.2), using the package CoxBoost (version 2.0) for the boosting approach.

### .632+ prediction error curve estimates

Although prediction performance of the fitted subdistribution hazard model is usually evaluated on a separate set of patients, this reduces the number of observations available for model fitting, decreasing the power to identify important genes. Therefore, we used all available patient data to fit the model. To evaluate the prediction performance for new patients, we employed a resampling technique. Briefly, 500 bootstrap data sets were generated, each containing a random sample of 0.632n patients of the original data [19]. Model development for the Fine-Gray model was performed for each resampling data set, including 10-fold cross-validation. The prediction performance was evaluated by “bootstrap 0.632+” estimates; the latter provides reliable estimates of prediction performance for new patients in a time-to-event setting [10, 19]. The Brier score, i.e. the squared difference between the predicted probability that a cardiovascular event will occur and the true status, was calculated and tracked over time, resulting in prediction error-curve estimates. In particular, the Brier score was chosen as a performance measure, as it simultaneously evaluates discrimination and calibration, and the Brier score difference between two models indicates the difference in precisions (see [20], for example). Thus, when comparing the Brier score of a model including gene expression model to the Brier score of some benchmark approach, the difference directly indicates by what amount the predicted probabilities of suffering a cardiovascular event up to some specific time get closer to the true patient status. The Aalen-Johansen estimator of the cumulative incidence, a performance reference that does not employ covariate information, was used as a null model. Furthermore, a Fine-Gray regression model was fitted that contains only clinical covariates age and prior cardiovascular events.

### RNA preparation

Whole blood specimens were collected in 2 × 2.5 ml PAXgene ^*TM*^ tubes from each subject, incubated at room temperature for 3 h to ensure complete lysis, and then stored at <80 degree C. RNA was extracted from whole blood using the PAXgene ^*TM*^ Blood RNA System (PreAnalytiX GmbH, Belgium), following the manufacturer’s instructions. The quality of the purified RNA was verified on an Agilent 2100 Bioanalyzer (Agilent Technologies, Palo Alto, CA). RNA concentrations were determined using a GeneQuant II RNA / DNA Calculator (Pharmacia).

### Microarray processing

Each RNA sample was amplified using the MessageAmp II aRNA kit (Ambion, Austin TX), using 1 *μ*g of total RNA according to the manufacturer’s instructions. In addition, Universal Human Reference RNA (Stratagene) was amplified and pooled as a reference for all hybridizations. cDNA microarrays were produced and processed essentially according to the Stanford protocol described by Eisen and Brown [21]. Approximately 38,000 annotated genes (Human Unigene Set - RZPD 3.1) from the RZPD (Resource Center and Primary Database, Berlin, Germany) were obtained as bacterial stocks, and amplified by polymerase chain reaction (PCR). PCR products were purified and transferred into 384-well plates. Printing was performed on aminosilane-coated slides (CMT-GAP II Slides, Corning, NY), using a Qarray2 arrayer from Genetix Ltd. (http://www.genetix.com/en/home/index.html). Post-processing was performed according to the Stanford protocol (http://brownlab.stanford.edu/). Hybridizations were performed in the presence of an equal amount of amplified reference RNA (Stratagene, La Jolla, CA, USA) as published [22]. All other steps, including hybridization, were performed following the Brown protocol. Signal intensities of the hybridized slides were quantified using an Axon 4000A scanner in combination with the GenePix Pro 6.0.1.17 image processing software (Axon Instruments, Union City, USA). After background subtraction, the log2 of the medians of the pixel intensity ratios Cy5/Cy3 within a spot were used to measure the expression of a gene relative to its abundance in the Universal Human Reference RNA. The Locally Weighted Scatterplot Smoothing (LOWESS) algorithm with a smoothing parameter of 0.2 was applied to adjust for the intensity-dependent bias between the two fluorophores [23]. Print-tip effects were corrected by a block-wise normalization procedure [24]. The Genedata Expressionist Refiner Pro 4.0 software (Genedata AG, Basel, Switzerland) was used for quality control.

### Quantitative RT-PCR

Quantitative RT-PCR experiments were performed using the Roche real-time PCR master mix (Lightcycler 480 Probes Master) in combination with the Roche Universal Probe Library (UPL) assays. All measurements were performed using the Roche Light Cycler 480 (Roche). Ribosomal protein L32 (RPL32) was used as a housekeeping gene (5’ccaccgtcccttctctctt3’; 5’gggcttcacaaggggtct3’) to quantify the expression of ITGA2B (5’gagacacccatgtgcagga3’; 5’agctggggcacacatacg3’), and PF4 (5’agcctggaggtgatcaagg3’; 5’gaagaccacctcccaggtc3’); all measurements were carried out in triplicates. Negative controls included a sample without reverse transcriptase and a sample without template. The regulation of genes was calculated using the normalized data derived from the relative quantification analysis.

## Results

### Patient survival

Informed consent and blood samples were obtained from 324 patients suffering from ESRD; three samples were discarded due to insufficient RNA quality. For the remaining 321 patients (Table 1), all relevant data were collected over a 2-year observation period. The mean age was 66 years (61 % male; 39 % female). As predicted from other studies, the one-year mortality in this population exceeded 15 %. Within follow-up, 30 deaths with and 71 deaths without prior cardiovascular event were observed. A later update of the endpoint information resulted in 39 deaths with prior cardiovascular event (three patients reclassified from no event and two from death without prior cardiovascular event), and 48 patients without prior cardiovascular event (11 reclassified from no event and 14 from death with prior cardiovascular event).

### Prediction performance using multivariable Fine-Gray regression

For a first analysis we consider separate Cox proportional hazards models for each of the 26323 features obtained from a cDNA microarray (after quality control and preprocessing), adjusted for age and prior cardiovascular events. Fitting Fine-Gray models [10] for directly assessing effects on the cumulative incidence of death with prior cardiovascular events, results in 236 genes with false discovery rate [25] below or equal to 5 %. While such a set could also be subjected to post processing steps, such as pathway analysis, we are primarily interested as to whether already the original data analysis can point out genes that provide insight into disease etiology. Correspondingly, we turned to an approach that could provide automated selection of markers in multivariable regression models. Specifically, we used componentwise likelhood-based boosting, as it is available for Fine-Gray regression modeling [10]. Adjusting for age and prior cardiovascular events, selection of the number of boosting steps (which determines the size of the signature) by 10-fold cross-validation, resulted in a signature comprising 12 microarray features. Of these, six were found to be rather stably selected, i.e. in at least 20 % of resampling data sets when repeating the signature building approach, as described subsequently (names indicated by boldface in Table 2). There was no overlap between the genes identified using this model and genes identified from a Cox regression model linking genes to “death without prior cardiovascular event”, suggesting that all genes identified from Fine-Gray regression are directly related to the occurrence of cardiovascular events.

**Table 2:** Inclusion frequencies for microarray features selected in at least 20 % of subsampling data sets by any of the approaches (sh: Fine-Gray regression; csh: cause-specific hazard model)

Feature	Original endpoint						Updated endpoint						Min	Max
	All observations				w/o outlier		All observations				w/o outlier			
	Multi		Uni		multi		Multi		Uni		multi			
	sh	csh	sh	csh	sh	csh	sh	csh	sh	csh	sh	csh		
**H57987**	0.30	0.38	0.31	0.50	0.33	0.40	0.31	0.37	0.41	0.50	0.33	0.38	0.30	0.50
**BX094448**	0.22	0.22	0.32	0.29	0.17	0.15	0.13	0.15	0.14	0.23	0.08	0.10	0.10	0.32
R36623	0.11	0.10	0	0.12	0.10	0.09	0.25	0.28	0.01	0.29	0.25	0.28	0	0.29
**R88065 (VPS72)**	0.23	0.20	0.10	0.17	0.27	0.25	0.07	0.10	0.11	0.15	0.10	0.13	0.07	0.27
BX104205	0.19	0.18	0.03	0.04	0	0	0.36	0.33	0.19	0.19	0	0	0	0.36
BX100481	0.14	0.10	0.05	0.01	0.09	0.06	0.26	0.24	0.12	0.04	0.19	0.17	0.01	0.26
BM918155	0	0	0	0	0	0	0.27	0.28	0.13	0.01	0.31	0.32	0	0.32
R00274	0.03	0.03	0.33	0.33	0.04	0.05	0.01	0.01	0.16	0.18	0.01	0.02	0.01	0.33
**BX281671 (ITGA2B)**	0.19	0.23	0.03	0.12	0.21	0.27	0.02	0.02	0	0.01	0.03	0.04	0	0.23
**R10279**	0.07	0.05	0.11	0.18	0.06	0.04	0.11	0.10	0.11	0.22	0.04	0.04	0.04	0.22
AA027034	0.04	0.03	0.17	0.05	0.04	0.03	0.04	0.04	0.25	0.07	0.04	0.04	0.03	0.25
AF086244	0	0	0	0	0	0	0.14	0.13	0.05	0.08	0.20	0.20	0	0.20
AA001661	0.04	0.03	0.16	0.22	0.04	0.03	0.01	0.01	0.06	0.17	0.01	0.01	0.01	0.22
**R06860**	0.21	0.11	0	0.03	0.17	0.09	0.01	0.01	0	0	0.01	0.01	0	0.21

Minimum and maximum inclusion frequencies are given in the two rightmost columns. Names of microarray features contained in the original signature are indicated by boldface

Among these there is ITGA2B (BX281671), a component of the platelet glycoprotein (GP) IIb/IIIa complex that mediates adhesion to extracellular matrix proteins and activation of platelets. While ITGA2B (integrin alpha 2b; CD41) mutations cause Glanzmann thrombasthenia [26], activation of the glycoproteins IIb/IIIa complex triggered by conformational changes plays a central role in thrombus formation and acute myocardial infarction [27]. Quantitative RT-PCR (RT-qPCR) was performed to validate the microarray data. Using RPL32 as a housekeeping gene [28], RT-qPCR revealed an increasing risk with higher levels of ITGA2B in a Fine-Gray model for this gene (*p* = 0.050). We also considered Platelet Factor 4 (PF4), as another platelet-specific protein [29], which was not represented on our microarray, but found no effect (*p* = 0.610).

Notable, in the ordered list of univariate *p*-values, ITGA2B occupies only position 43, i.e. would probably not have been considered for further analysis, despite its potential biological relevance. Therefore, one question is whether, such a gene from the middle field of the list of genes significant according to univariate analysis *p*-values can more reliably be identified by a multivariable approach for a short list of important genes. Another candidate for such considerations might be VPS72 (R88065), vacuolar protein sorting 72 homolog, which has recently been associated with thrombopoietin-mediated maintenance of hematopoietic stem cells [30].

**Fig. 2:**
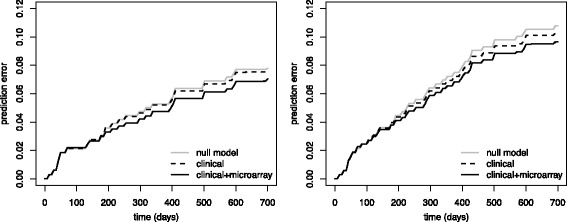
Prediction error curves..632+ prediction error curve estimates for the microarray signature for the original (*left* panel) und the updated endpoint information (*right* panel), considering an Aalen-Johansen estimator (which doe not use any patient information), and a purely clinical model as a benchmark

As a first step for judging the evidence supporting these genes, we considered prediction performance on new data, as evaluated by.632+ prediction error curve estimates [10]. Models utilizing clinical data were better than the null model not using any covariate information (left panel of Fig. 2). However, the Fine-Gray model combining clinical and microarrays performed superior to the clinical model alone, supporting the hypothesis that the gene expression data contains prognostic value beyond the clinical covariates. To provide an indication of whether the difference is statistically significant, we calculated the integral of the difference between the respective resampling cross-validation error estimates, which are the basis for the.632+ estimates. A Wilcoxon test indicated that this difference is significantly different from zero across resampling data sets (*p* < 0.001). To furthermore check whether there might be an interaction between clinical an microarray covariates, we separately extracted the linear predictors for the clinical and the microarray covariates, and entered them as covariates into a new Fine-Gray regression model that included an interaction term between the two. The latter term was found to be significant (*p* = 0.039), indicating that the clinical+microarray model might be improved further by incorporating interaction terms, but we will not pursue this in the following.

Prediction performance might be problematic as a sole criterion for judging prognostic signatures. To illustrate this, the right panel of Fig. 2 indicates the prediction performance obtained when applying the componentwise likelihood-based boosting approach for the updated endpoint information. While there seems to be some decrease of prediction performance relative to the null model, the overall picture of the clinical model performing better than the null model, and the combined model performing even better, stays similar. Still, a Wilcoxon test no longer indicated a significant difference between the clinical and the clinical+microarray model (*p* = 0.268). The boosting approach for the latter on the full data set now selects a prognostic signature of 19 genes, which contains only three of the microarray feature (BX094448, H57987, and R10279) selected by boosting for the original endpoints. Notably, ITGA2B and VPS72 are absent.

This calls for a different set of tools for judging whether identification of ITGA2B and VPS72 was just an artifact. Before introducing such tools for stability analysis based on resampling inclusion frequencies, we use the inclusion frequencies for identifying potential outliers that might affect selection of genes for a prognostic signature, due to artificial correlation.

### Identifying potential outliers affecting selection

To quantify stability, we performed signature selection repeatedly in 10,000 subsamples half the size of the original data, drawn without replacement. Along the lines of stability selection [15], boosting was performed in each of these subsampling data sets with a fixed number of boosting steps, i.e. a fixed level of model complexity. Specifically, 100 boosting steps were performed. In theory, this would allow for up to 100 signature genes (as one non-zero coefficient of the regression model can be added or updated in each boosting step). However, on average only 11 genes were selected, i.e. the regression parameter of each of of these genes received several updates. To mimic similar selection, *p*-values from univariate models, i.e. per gene, were also calculated in each of the subsampling data sets, and the 11 microarray features with the smallest *p*-values were considered as selected. Resampling inclusion frequencies were obtained by determining for each gene the proportion of subsampling data sets where the respective gene was selected to be part of the signature.

To investigate whether there might be observations, such as outliers, that particularly influence signature selection, we consider a set of genes with at least moderate inclusion frequency. Specifically, there are 15 genes that are selected in at least 10 % of the subsampling data sets by the boosting approach for the original endpoint information (most frequently selected genes shown in Table 2). For these we calculated pairwise cross-tables with respect to signature inclusion. The rationale behind this is that correlation structure may lead to strong effects of inclusion of one covariate in a regression model conditional on the in- or exclusion of another covariate [14]. While such behavior of automated variable selection approaches might be desirable for avoiding groups of genes in signatures that essentially carry the same information, some of this might also be due to few influential observations such as outliers.

**Fig. 3:**
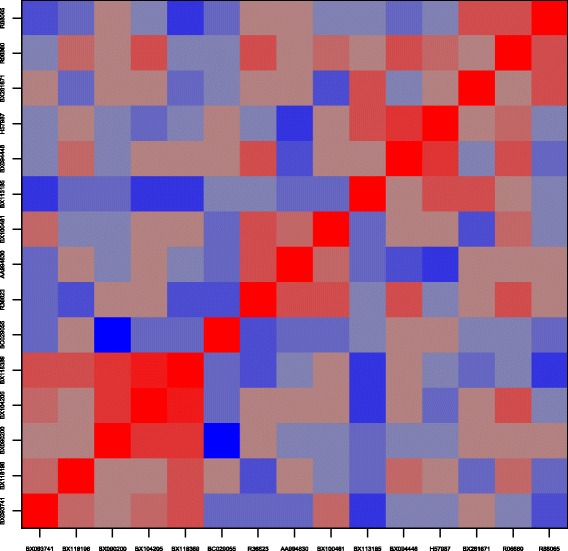
Joint selection. Odds ratios of joint selection for microarray features with inclusion frequency larger or equal to 0.1. Blue color indicates odds ratios <1, i.e. alternative selection, red colors >1, i.e. joint selection, with more intense color indicating more extreme effects

**Fig. 4:**
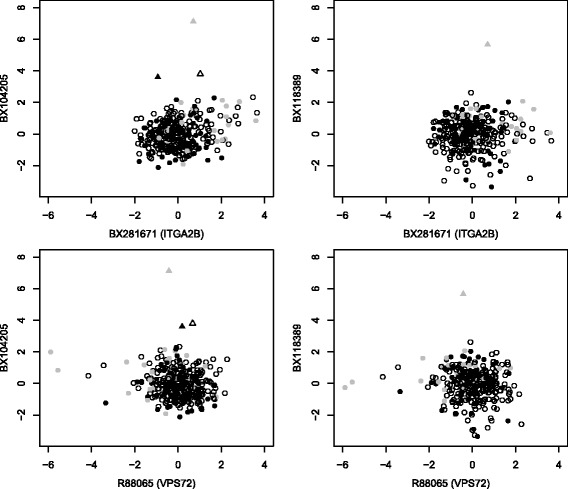
Outlier detection. Scatter plots of standardized log2 expression values of some microarray features with strong conditional signature non-inclusion. The three observations considered as outliers for later analysis are indicated by triangles. Censored observations are indicated by hollow symbols, deaths after cardiovascular event by filled grey symbols, an deaths without prior cardiovascular events by filled black symbols

Figure 3 indicates the pairwise odds ratios from the pairwise cross-tables. There seems to be a cluster of five genes in the bottom left corner that facilitate inclusion of each other, with a core set of three microarray features (BX090200, BX104205, and BX118389). Presence of one of these three microarray features frequently seems to coincide with exclusion of other microarray features, as indicated my many darkly colored fields in the corresponding rows and columns. To investigate correlation of these three features with ITGA2B (BX281671) and VPS72 (R88065), which might lead to potential exclusion, we consider pairwise scatter plots of (standardized) log2 expression values in Fig. 4. There are three observations with rather large value for BX104205. One of these three also has a rather large value for BX118389. For both microarray features, these extreme values, which might be outliers, are not in line with a potential linear trend between BX104205/BX118389 and BX281671/R88065 respectively. Therefore, we consider exclusion of these three observations for subsequent stability analysis.

### Impact of modeling choices on stability

For stability analysis, we consider the following modeling choices: (1) The type of bioinformatic approach for signature selection (per-gene Cox models vs. boosting for fitting a multivariable model), (2) exclusion of (the three) potential outliers vs. analysis using all observations, (3) the type of the endpoint approach, Fine-Gray regression vs. Cox regression for cause-specific hazards, and (4) using the original endpoint vs. the updated endpoint information. Many of the possible 16 combinations were performed in each of the 10,000 subsampling data sets.

Table 2 shows the inclusion frequencies for all genes that received an inclusion frequency of at least 20 % in at least one of the considered modeling choices. Generally, the inclusion frequencies are considerably smaller compared to what might be expected in a low-dimensional regression setting, where variables can be selected more reliably, but comparable to other applications to gene expression data [14]. There are rather stably selected microarray features, such as H57987, which are identified regardless of the specific modeling choice. For other genes the inclusion frequencies vary widely. The choice between Fine-Gray regression (indicated by “sh”) and Cox regression for cause-specific hazards (indicated by “csh”) leads to changes in inclusion frequencies of up to 20 %. There is no clear tendency towards larger inclusion frequencies for one of the two modeling approaches, but some genes seem to be selected more stably when using Fine-Gray regression, e.g. R06860, and some by cause-specific modeling, e.g. ITGA2B (BX281671). For the latter gene, choice of the endpoint seems to be more important for stable selection than choice of the specific regression model. This this is also the case for VPS72 (R88065). For these genes, use of cause-specific hazard boosting together with the original endpoint and exclusion of the potential outliers drastically improves selection stability (from less than 1 to 27 % BX281671). This is remarkable, as the different modeling approaches do not directly optimize for selection stability, prediction performance being the primary criterion.

To more formally evaluate the effect of modeling choices, we fit a binomial regression model for the per-gene inclusion frequencies conditional on the combinations of different modeling choices. This model includes main effects for the four modeling choices above, and two-way interactions (Up to four-way interactions were considered in preliminary analyses but turned up no strong effects beyond two-way interactions). The results for ITGA2B are shown in Table 3. Using the original vs. the updated endpoint information has the largest impact. Use of per-gene models vs. boosting for multivariable modeling is seen to have an impact comparable to that of excluding the three potential outliers. The specific type of regression model (cause-specific vs. Fine-Gray) is seen to have the least impact when combined with the boosting approach. For the per-gene approach, the type of hazard model seems to have a much larger impact (term “sh × univariate” in Table 3). One explanation might be that there is much stronger competition for the top 11 spots in the list of *p*-value sorted genes, and changes in the hazard model shift the balance between genes. In contrast, the boosting approach is directly optimized for picking only few genes, leaving room for some genes that might only be relevant for a specific hazard setting.

**Table 3:** Binomial regression model for signature inclusion of microarray feature BX281671 (ITGA2B) in different modeling approaches (sh: Fine-Gray regression; csh: cause-specific hazard model)

Term	ITGA2B			All features	
	estimate	*p*-value		sig -	sig +
Intercept	−3.82	<0.001		-	-
sh vs. csh	−0.16	0.013		24	16
Outlier excluded	0.58	<0.001		29	19
Original endpoint	2.63	<0.001		**30**	**23**
Univariate model	−0.98	<0.001		**31**	**23**
sh × outlier excluded	−0.01	0.798		0	0
sh × original endpoint	−0.12	0.056		19	17
sh × univariate	−1.27	<0.001		31	25
Outlier excluded × original endpoint	−0.43	<0.001		19	11
Original endpoint × univariate	0.15	0.055		21	14

The numbers of significant effects (5 % level after Bonferroni correction) for all 58 microarray features with inclusion frequency larger or equal to 0.1 in any of the approaches are indicated in the two rightmost columns, separately by positive and negative signs

The impact of the modeling choices on selection with respect to all genes is assessed by fitting a separate binomial regression model for the signature inclusion of each gene that has an inclusion larger or equal to 0.1 for any of the modeling approaches. After Bonferroni correction, the endpoint update and the type of approach for signature selection are seen to have an effect on the largest number of genes (highlighted by boldface in the two rightmost columns of Table 3). Similar to the effect seen for ITGA2B, the per-gene approach seems to result in decreased stability, as compared to the multivariable approach (term “univariate” in Table 3).

**Fig. 5:**
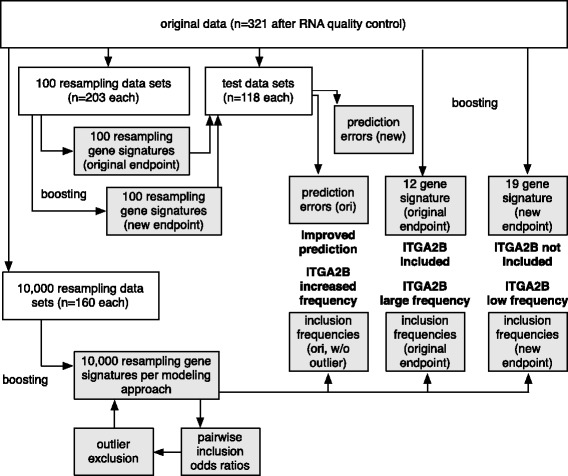
Data and analysis flow. Use of original data and resampling data sets for different analyses, specifically, for obtaining signatures, estimating prediction performance, identifying outliers and judging stability of selection, exemplarily illustrated for ITGA2B

The overall contribution of different analysis components for more comprehensively judging the effects of genes is exemplarily illustrated for ITGA2B in Fig. 5. Using boosting for obtaining two gene signatures, for the original endpoint and the new endpoint respectively, already indicated that detection of ITGA2B might considerably depend on model choice. Still, this result might have been due to random variability, as signatures are known to be highly unstable [9]. As indicated above, we leveraged several resampling-based building blocks for getting more firm conclusions. Estimates of prediction error indicated that there actually might be prognostic information in the gene expression measurements, warranting further investigation of gene signatures. Pairwise resampling inclusion frequencies allowed for detection of potential outliers, expanding model choices by outlier exclusion. Finally, per gene inclusion frequencies allowed to judge the effect of the different modeling choices, in particular concerning the original result on the model choice effect on selection of ITGA2B.

## Discussion

The high cardiovascular morbidity and mortality in patients with ESRD and chronic maintenance-hemodialysis remains an unsolved problem. The grim prognosis of ESRD, particular during the first year, has not significantly changed over the past years [31]; despite significant technological advances and use of extensive resources, dialysis patients suffer a >20 % annual mortality rate in most countries [32]. It is remarkable that even young patients, typically not at risk for cardiovascular disease, face a dramatic increase in mortality once exposed to hemodialysis [33]. Another concerning aspect is the complete failure of lipid-lowering drugs, typically powerful agents to combat cardiovascular disease, to improve the survival of hemodialysis patients [34, 35]. Moreover, anticoagulation and platelet inhibitors negatively affect survival of hemodialysis patients; in a large meta-analysis, patients exposed to warfarin, clopidogrel, and/or aspirin had significantly increased mortality rates [36, 37]. These observations suggest that the prevalence of cardiovascular disease is only partly explained by conventional risk factors, and that the mechanisms causing cardiovascular disease and death in hemodialysis patients differ from other populations [38]. This urgent medical problem motivated us to tackle development of a prognostic gene expression signature for identifying targets to predict or treat cardiovascular disease in hemodialysis. We speculated that the gene profile of peripheral blood cells might mirror some of the pathological changes that lead to cardiovascular disease in this population.

Prediction performance of signatures developed by a multivariable regression approach showed improved prediction performance compared to a purely clinical model, indicating that there might be some relevant prognostic information in the gene expression measurements. Well aware that prognostic signature development might nevertheless be highly unstable due to the small number of events [9], we developed a strategy for not only identifying a signature, but quantifying stability and the influence of different modeling choices on stability. While selection stability will generally be not as good as in low-dimensional applications [14], changes in stability might nevertheless be useful for guiding modeling.

Our approach is based on resampling inclusion frequencies, and not only allows to quantify stability, but also helped to flag potential outliers that might affect signature building. The effect of three such potential outliers on selection stability was seen to be on a level similar to the impact of choosing a specific bioinformatic approach for gene selection. The largest impact on stability was seen for an update of endpoint information, which might be frequently performed in cohort studies with long follow up, i.e. there is a high likelihood that in many studies signature stability might be affected by issues with endpoint information.

A disadvantage of such a resampling approach for stable selection of genes is that it no longer results in a gene signature (as originally provided by the underlying multivariable regression technique), but just a list of genes with resampling inclusion frequencies. Still, using a cutoff on the latter, a set of genes could be selected and combined into a rule for risk stratification, e.g. by weighting the genes according to the average of estimated regression coefficients from the resampling data sets, or by a simpler scoring rule where 1 point in a risk stratification score is assigned (or deducted) for each over-expressed gene.

One particular gene whose identification was affected by the specific modeling choices was ITGA2B. Our strategy allowed us to better judge whether this gene might truly be associated with cardiovascular events. In addition, such effects also are biologically plausible. In myocardial infarction, the platelet glycoprotein (GP) IIb of the IIb/IIIa complex is activated, and accelerates platelet aggregation and thrombosis by acting as a receptor for fibrinogen; conversely, inhibition of the GP IIb/IIIa complex has been shown to ameliorate the outcome of cardiac ischemia [27, 39]. Our results now indicate that ESRD patients with an increased ITGA2B expression after a two-day dialysis-free interval have an increased risk to develop a cardiovascular event over an observation period of two years. The surface of resting platelets typically contains 500 to 800 GP IIb/IIIa complexes [40]. Our results suggests that GP IIb/IIIa complexes accumulate on platelets of hemodialysis patients with high cardiovascular risk. Accelerated GP IIb/IIIa-mediated cross-linking of platelets during ischemia may facilitate rapid thrombus formation, and perhaps contributes to the high frequency of sudden cardiac death and unfavorable outcome after myocardial infarction in ESRD patients. Platelet activation and the formation of platelet-leukocyte aggregates during hemodialysis have been utilized to determine the biocompatibility of a renal replacement modality for several years [41]. However, more recently, the presence of platelet-monocyte aggregates has been linked to an increased cardiovascular risk in patients with ESRD irrespective of the dialysis modality [42].

A limitation of the present study is that there is no external data for validation of increased risk. While the proposed.632+ resampling approach repeatedly performs signature selection and risk assessment on separate subsets of the data to estimate performance on new data, this cannot fully replace real external validation data. For example, the latter will typically exhibit some structural differences, which cannot be anticipated by the resampling approach. Also, while biologically plausible, results were seen to be strongly affected by the different modeling choices. Also, similar to other gene expression applications, stability of gene selection generally was rather low, making it difficult to reliably pinpoint single genes for subsequent validation. Therefore, additional studies are needed to validate that increased expression of platelet markers predicts an increased risk for cardiovascular events in ESRD patients.

## Conclusions

ESRD patients experience a high mortality rate due to cardiovascular events that is poorly explained by conventional risk factors. Gene profiling in combination with clinical parameters revealed that abnormal expression of platelet proteins might predict an increased risk for cardiovascular disease. Oral GP IIb/IIIa antagonists used in the setting of acute myocardial infarction have generally been disappointing, and a meta-analysis suggested a higher mortality for ESRD patients treated with clopidogrel and/or aspirin [43]. However, our data suggest that a preemptive therapy targeted against aberrant platelet activation may be beneficial to ESRD patients at risk for cardiovascular disease. Thus, it will important to evaluate in prospective studies whether measures that prevent platelet activation decrease the incidence of cardiovascular events in ESRD patients.

The proposed strategy for analyzing prognostic signature selection stability allowed us to precisely quantify the impact of modeling choices on the stability of marker identification. Thus we suggest routine use also in other applications to prevent analysis-specific results, which are unstable, i.e. not reproducible.
